# Mental Health Benefits of Listening to Music During COVID-19 Quarantine: Cross-Sectional Study

**DOI:** 10.2196/46497

**Published:** 2024-12-16

**Authors:** Xuechang Xian, Xiaoran Zhang, Danhe Zheng, Yanlin Wang

**Affiliations:** 1 Tourism and Historical Culture College, Zhaoqing University, Zhaoqing, China; 2 Faculty of Social Sciences, University of Macau, Macau SAR, China; 3 School of Public Administration & Law, Fujian Agriculture and Forestry University, Fuzhou, China; 4 Faculty of Law, University of Macau, Macau SAR, China; 5 Department of Music and Dance, Jingdezhen University, Jingdezhen, Jiangxi, China; 6 Rehabilitation Industry Institute, Fujian University of Traditional Chinese Medicine, Fuzhou, Fujian, China

**Keywords:** COVID-19, quarantine, social connectedness, sense of security, mental well-being, cross-sectional study, contagion, treatment, music, security, mental health, questionnaire, China, intervention, relaxation, meditation, mental illness, stimuli, environmental

## Abstract

**Background:**

COVID-19 has posed a significant global threat to public health due to its high contagion risk and lack of effective treatment. While quarantine measures have been crucial in controlling the virus’ spread, they have also contributed to negative impacts on individuals’ mental health. Music listening has emerged as a potential coping mechanism, yet it remains unclear whether mental well-being varies across music preferences.

**Objective:**

This study examined individuals’ music-listening preferences in the context of COVID-19 quarantine and assessed the mediation pathways linking 5 types of music to mental health levels, mediated by perceived social connectedness as well as sense of security.

**Methods:**

A web-based survey was conducted among people with quarantine experience in September 2022, in mainland China. A total of 712 valid questionnaires were returned and 596 samples were finally included in our study for mediation analysis.

**Results:**

The results revealed that the vast majority (596/623, 96%) of respondents had music-listening experiences during the COVID-19 quarantine, with pop music emerging as the most popular preference among respondents, while quyi was the least listened-to genre. Additionally, listening to music across 5 different genres appeared as a significant parameter indirectly linked to mental health through perceived social connectedness. Specifically, engaging with quyi was associated with higher levels of perceived social connectedness and sense of security, which in turn correlated with improved mental well-being. Conversely, individuals listening to jazz reported lower social connectedness and sense of security, which was subsequently linked to increased mental health problems. The potential reasons for these findings and implications are discussed.

**Conclusions:**

This study significantly contributes to the understanding of the mechanisms behind music-listening preferences in stressful environments. Specifically, our findings highlight the mediating roles of perceived social connectedness and sense of security in the relationship between music preferences and mental health outcomes during the quarantine period. These insights provide valuable guidance for developing interventions that use music to enhance mental health, thereby broadening the scope of studies on environmental stimuli and their impact on mental well-being.

## Introduction

### Overview

COVID-19 has threatened the world since 2020. The impact of the public health crisis is significant because of the highly contagious nature of the virus and the lack of an effective treatment. In order to contain the spread of COVID-19 effectively, many countries have required citizens to stay at home and refrain from socializing or, at the early stage of the COVID-19 pandemic, enforced mandatory isolation for citizens at risk of illness [1]. China was the first country to implement nationwide quarantine policies in response to the COVID-19 outbreak. Unlike many other nations that have adopted “co-existence” strategies—gradually easing restrictions, improving therapeutics, and relying on voluntary isolation [2]—it has maintained a strict “COVID-19 elimination” approach, with mandatory quarantine measures (ie, home or institutional quarantine) at its core. For example, mass quarantine in domestic facilities (eg, stay-at-home quarantine) is enforced when a region is found to have a high risk of COVID-19 transmission and a high rate of community infection [3]. Such measures aim to block the spread of diseases in the community by physically isolating the infected individuals from the uninfected individuals. Conversely, collective quarantine in professional medical facilities (ie, institutional quarantine) is used for individuals identified as having had close contact with COVID-19 patients or those returning from high-risk regions [4]. All these quarantine measures were found to be effective in reducing the risk of disease transmission at the early stage of the outbreak.

However, the cost of quarantine is substantial. The restriction of freedom of an uncertain duration, separation from families or friends, disruption of people’s daily life, disease uncertainty, and the resulting boredom could have all contributed to mental problems such as anxiety and depression. At the onset of the COVID-19 outbreak, many people in quarantine reported high levels of stress, anxiety, or depression and low levels of life satisfaction [2,5-7]. Therefore, it is necessary to understand the public’s mental health issues in the context of the mandatory quarantine and identify solutions to minimize the negative psychological impacts associated with home or institutional quarantine and, thus, support the self-management of well-being across the quarantine period.

Music is ubiquitous in people’s daily lives, and listening to music is a popular leisure activity that benefits individual well-being. During the COVID-19 outbreak, music was widely used by individuals as an emotional resource to sustain them through difficult times. A growing body of research has examined the effect of music on individuals’ well-being during COVID-19 [8,9], with 1 study reporting that people tend to use music for managing emotions, regulating distress, and reducing symptoms of depression [10]. Although the positive relationship between music and mental health has been well documented (eg, [11]), there is a need for a more detailed investigation into the heterogeneous effects of various music genres. The common music genres appearing in digital media channels include classical, pop, folk, quyi (a type of Chinese traditional opera), and cartoon music [12]. In recent years, rock, rap, jazz, hip-hop, and R&B music have become increasingly popular in China [13]. Despite the emergence of increasingly diverse music as an emotional resource, little research has been conducted to understand individuals’ music-listening preferences and explore their associations with mental outcomes in the context of COVID-19 quarantine. In other words, it is unclear whether the association between music listening and mental well-being is unique to specific music preferences or is shared by all music types.

Music genres are characterized by different features. For example, pop music, in contrast to other more niche genres, such as rock, rap, and jazz, tends to appeal to a broader and diverse audience [14]. The lyrics in pop music are characterized by themes of personal growth, relationships, autonomy, identity, and peer acceptance [15]. These features of pop music facilitate social communication among subgroups of peers [16]. As a result, listening to pop music might be helpful for increasing feelings of connectedness with others because it provides an opportunity for social interaction with a large fan group that could prompt the formation of a common identity [17]. Furthermore, the feeling of support from peers might ease the loneliness of isolation from families and friends. For example, pop music listeners often share music on social media in order to express themselves and communicate with their peers, thus likely making themselves more socially active and connected and improving their mental well-being.

Quyi, a traditional form of Chinese music, is often characterized by ballad singing and storytelling [18]. Quyi is part of everyday life for the common people in China, who obtain greater feelings of contentment through the close atmosphere between the audience and performers in quyi than with rock, rap, or jazz. Such a type of music is able to enhance feelings of social connectedness due to its familiarity and the nature of its storytelling. Indeed, in health promotion settings, storytelling interventions have been widely used as an effective tool to strengthen social connectedness by stimulating memories [19,20]. This increased feeling of social connectedness likely leads to greater life satisfaction and, subsequently, better mental status.

Rock music is often associated with themes of rebellion, freedom, and self-expression, making it particularly resonant with people who are navigating their identities and emotions [21]. Its energetic rhythms and powerful lyrics can serve as a cathartic outlet for young people, helping them process emotions such as anger or frustration [22]. Studies have suggested that listening to rock music can provide a sense of empowerment and community, as it often addresses topics such as personal struggles and societal issues [23,24].

Rap music is known for its strong rhythmic elements and lyrical storytelling, often exploring themes such as social injustice, personal perseverance, and community pride [25,26]. For many adolescents, especially those from marginalized communities, rap serves as a powerful medium for expressing their experiences and challenges. Studies have indicated that engaging with rap music can develop a sense of identity and resilience [27,28]. This may make it a valuable resource for emotional processing and maintaining mental well-being.

Jazz music, characterized by its improvisational nature and complex structures, can positively influence cognitive development and emotional well-being [29,30]. For young people, engaging with jazz—whether through listening or playing—provides a sophisticated way for emotional expression. Its intricate rhythms and melodies encourage deep, active listening, which can be both intellectually stimulating and emotionally soothing [31]. During the COVID-19 pandemic, the therapeutic qualities of jazz, particularly its complexity and calming rhythm, may have made it an effective tool for promoting mindfulness and mental clarity, providing a sense of relaxation amidst the challenges of the time.

### Objectives and Hypotheses

**Figure 1.**
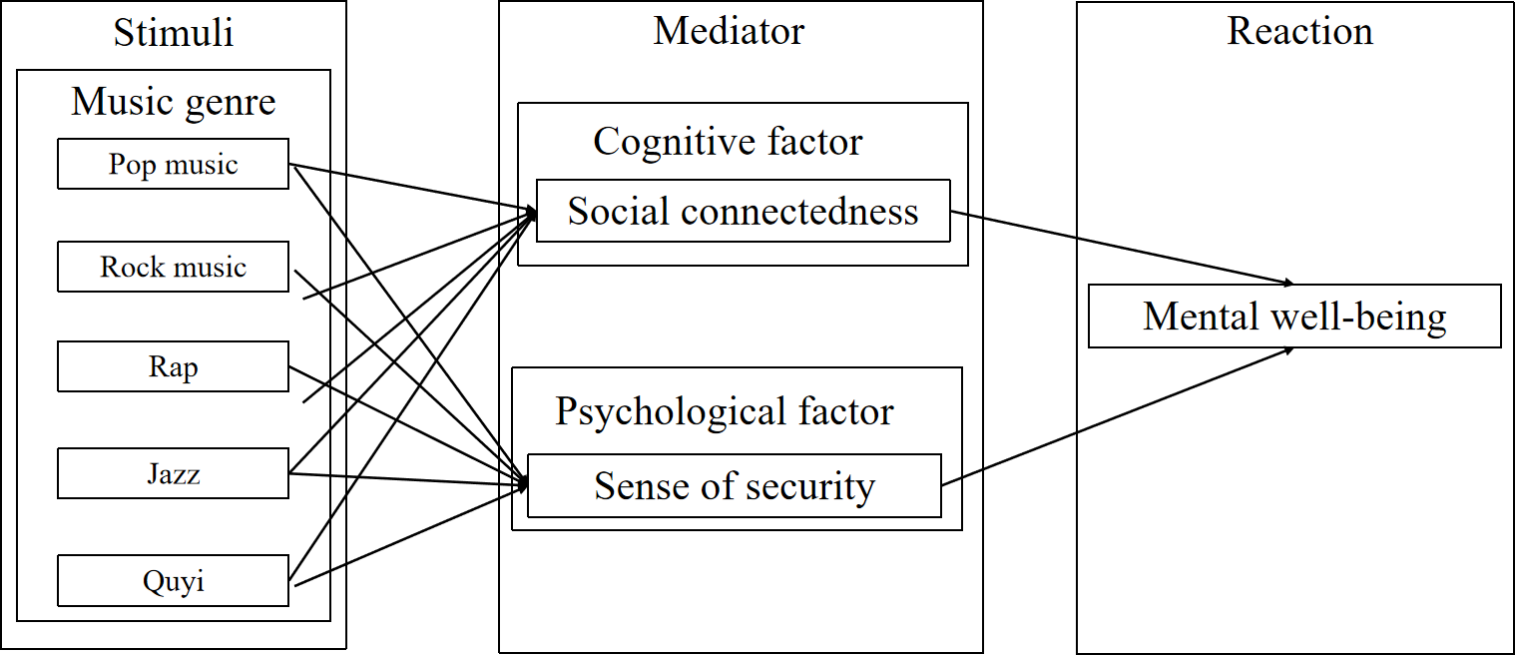
Conceptual framework.

This study aims to understand the music-listening behaviors of people during the COVID-19 quarantine and examine how the heterogeneity in music preferences impacts mental outcomes. To this end, a conceptual framework proposed by Miranda and colleagues [32] was used to guide this research. Miranda’s framework, particularly the focus on music preferences for negative themes and their impact on internalizing symptoms through mechanisms such as rumination or co-rumination, has provided a valuable conceptual foundation for our research. We extend this by exploring how distinct music preferences (ie, pop, rock, rap, jazz, and quyi) during COVID-19 quarantine affect mental well-being. Specifically, given the unique cognitive and psychological challenges posed by quarantine, our study builds on Miranda’s focus to examine how listening to music from different preferences can mitigate negative effects by fostering a sense of social connectedness and emotional security, ultimately influencing mental health. Our conceptual framework is presented in Figure 1.

Specifically, our proposed framework indicates that music preferences may influence mental disorders through cognitive and psychological processes. The cognitive influence on perceived social connectedness is particularly biased during quarantine due to the loss of freedom and suppression of expression. This suppression includes constraints on physical and emotional communication, limited face-to-face interactions, reliance on digital communication, and restrictions on social activities, all of which hinder authentic expression and impact social connectedness. Perceived social connectedness in health care refers to a subjective cognition related to an individual’s amount and quality of interpersonal relationships [33]. This concept reflects the closeness individuals perceive from others or society [34]. During the COVID-19 outbreak, people were unable to work or live as normal. In this context, interpersonal communication was constrained by social distancing and both stay-at-home and institutional quarantine measures, leading to issues such as loneliness [35]. Music listening could act as a replacement for social experience by creating a sense of the physical presence of others even when the individual is alone [36]. Therefore, the intensified social connectedness perceived by individuals due to music listening may enhance health outcomes such as positive mental health status.

Another path focuses on psychological influence. During COVID-19 quarantine, individuals may fear becoming unwell or being unable to cope with unexpected events, thus giving them a low sense of security. A sense of security is shaped by experiences, and the feeling of insecurity is often associated with the experience of being in a risky environment [37]. In terms of music, digital music listening might intensify individuals’ feelings of security by motivating them to establish a degree of control over their situation, thereby benefiting their mental health [38]. Based on reviews of past studies, several associations can be hypothesized:

Hypothesis 1: Engaging with different music preferences may have a relationship with the mental well-being of individuals under quarantine.

Hypothesis 2: Engaging with different music preferences may be associated with higher levels of perceived social connectedness, which in turn may improve mental well-being.

Hypothesis 3: Engaging with different music preferences may be linked to a better sense of security, which in turn may enhance mental well-being.

## Methods

### Overview

The analyzed data were collected in September 2022 in mainland China through a web-based survey conducted by Mengwen, a survey company specializing in market research. A convenience sampling method was employed to facilitate the data collection process. Participants were asked whether they had an isolation experience, such as institutional or home quarantine, within the last 14 days. Only those who provided a positive response could continue with the survey.

### Ethical Considerations

Ethical guidelines were strictly followed to ensure the protection of our participants’ rights and well-being. Informed consent was obtained from all participants before they began the questionnaire. The consent form detailed the study’s purpose, procedures, potential risks and benefits, confidentiality, and participants’ rights, including the voluntary nature of participation and the option to withdraw at any time without consequences. For participants under 18 years, the same information was provided to both the minors and their parents or guardians. Anonymity and confidentiality of responses were assured, and only those who agreed to the terms were allowed to proceed with the questionnaire. This study was approved by the research review board of Jingdezhen University.

### Measurement

The Patient Health Questionnaire-2 and Generalized Anxiety Disorder-2, as frequently used in the Health Information National Trends Survey studies in the U.S., were employed to assess mental well-being in this study for efficiency and alignment with established methodologies [39]. Specifically, respondents were asked how often they had been bothered by (1) little interest or pleasure in doing things; (2) feeling down, depressed, or hopeless; (3) feeling nervous, anxious, or on edge; and (4) not being able to stop or control worrying during the quarantine period. The participants responded to each item using a 5-point Likert scale (1=not at all; 5=always). All items were recoded into a scale with 1=always and 5=not at all, and then the scores were aggregated and averaged to indicate the individual’s level of mental well-being (mean 2.95 [SD 1.09]; Cronbach *α*=.88). Higher scores indicate better mental well-being of the respondent.

The independent variable, digital music-listening preferences (hereinafter referred to as music preferences), was measured by a series of questions derived from previous research [12]. Music preferences refer to an individual’s choices in digital music listening when under quarantine or similar situations. First, the respondents were asked whether they had listened to music via digital media in the past 14 days of quarantine (or a similar societal restriction). Individuals with prior music-listening experience were subsequently inquired about the particular genres they had listened to. The respondents were allowed to select more than 1 genre, and each genre selected was coded as 1; if the genre was not selected, it was coded as 0. The music genres included pop, rock, rap, jazz, quyi, and other music. All the responses were treated as dichotomous variables (0=no, 1=yes).

The mediators in this study included perceived social connectedness and sense of security, both of which were assessed based on responses to the 5-point Likert scale (1=strongly disagree to 5=strongly agree). Specifically, perceived social connectedness was measured by 6 items drawn from prior research by Lee and Robbins [40]. The respondents were given the following items: “Over the past 14 days, to what extent do you agree with the descriptions such as ‘I feel disconnected from the world around me’ and ‘I feel so distant from people’?” All the items were recoded into a scale (with 1=strongly agree and 5=strongly disagree), and then the scores were aggregated and averaged to reflect the individual’s perceived social connectedness (mean 3.04 [SD 1.11]; Cronbach *α*=.92), with higher scores indicating better perceived connectedness to society.

Sense of security was measured by 6 items adapted from previous research [41]. Sample items include “Over the past 14 days of quarantine, to what extent do you agree with the descriptions such as ‘I am always worried that my life will turn into a mess’ and ‘I feel that my life is full of uncertainty and unpredictability’?” All the items were recoded into a scale (with 1=strongly agree and 5=strongly disagree), and then the scores were aggregated and averaged to indicate the level of sense of security of the individual (mean 2.87 [SD 1.03]; Cronbach *α*=.9), with high scores suggesting a greater sense of security.

To ensure the relevance and clarity of the instrument for our specific context, we conducted a thorough review and selection process of items from the original scale. The selection of items was based on their relevance to the quarantine context and their ability to capture the core aspects of a sense of security. We used prior literature as a reference and consulted with experts in psychology and psychometrics to identify the most pertinent items, which were adapted and refined to ensure they accurately reflected the experiences and concerns of individuals during the COVID-19 quarantine.

During data analysis, the PROCESS macro for SPSS (IBM) was used to implement mediation analysis. To ensure that sociodemographic characteristics were taken into account in the regression equations generated by PROCESS—thereby mitigating potential confounding effects—we included them as control variables while defining the model. These covariates include age (coded as 1=18 y old or below to 7=60 y old and above), gender (1=male, 2=female), education (coded as 1=primary school education or below to 7=doctoral degree), household income (coded as 1=less than CNY ¥100,000 [US $13,772.21; CNY ¥1=US $0.14] per year to 3=more than CNY ¥200,000 [US $27,544.42] per year), and number of vaccines received (coded as 0=none to 4=four and above). The inclusion of the number of vaccines received was determined based on the premise that vaccination might influence mental health during the COVID-19 quarantine period [42].

## Results

Overall, a total of 712 people were eligible to participate in the survey. To ensure the reporting quality, only those questionnaires that were answered for at least 200 seconds (n=623) were included in our study for analysis.

### Descriptive Statistics

Table 1 presents the descriptive statistics of the sociodemographic variables. The results showed that 92.1% (574/623) of the participants ranged from 18 to 40 years old, and 54.9% (342/623) reported themselves to be female. With regard to education level, 68.7% (428/623) received a bachelor’s degree or above. Over half of the respondents (323/623, 51.8%) had a yearly household income ranging from CNY ¥100,000 to ¥200,000. In addition, 72.4% (451/623) of the respondents had received 3 vaccines, and 30.2% (188/623) resided in East China, followed by 27% (168/623) in North China and 15.4% (96/623) in South China.

**Table 1.** Sample characteristics.

Variable	Participants, n (%)
Gender	
Male	279 (44.8)
Female	342 (54.9)
Others	2 (0.3)
Age	
Under 18 years old	13 (2.1)
18‐25 years old	211 (33.9)
26‐30 years old	225 (36.1)
31‐40 years old	138 (22.2)
41‐50 years old	32 (5.1)
51‐60 years old	2 (0.3)
60 years old and beyond	2 (0.3)
Education	
Primary school education and below	4 (0.6)
Secondary school education	17 (2.7)
High school education	53 (8.5)
Vocational school education	121 (19.4)
Bachelor’s degree	324 (52)
Master’s degree	73 (11.7)
Doctoral degree	31 (5)
Household income (CNY ¥)	
<100,000	148 (23.8)
100,000‐200,000	323 (51.8)
>200,000	119 (19.1)
Failed to disclose	33 (5.3)
Number of vaccines received	
None	3 (0.5)
One	14 (2.2)
Two	143 (23)
Three	451 (72.4)
Four and above	12 (1.9)
Residence	
North China	168 (27)
East China	188 (30.2)
Central China	82 (13.2)
South China	96 (15.4)
Southwest China	30 (4.8)
Northwest China	19 (3.05)
Northeast China	40 (6.4)

Our results indicated that around 96% (596/623) of respondents reported having listened to music during quarantine. Among the music preferences examined, pop music was the most popular genre, with 73.2% (436/596) of respondents reporting it as their preferred listening experiences during quarantine. This was followed by rap (35.4%, 211/596), rock (28.5%, 170/596), jazz (22.5%, 134/596), and quyi (17.6%, 105/596). Additionally, 7% (40/596) of the respondents indicated that they had listened to other types of music not specified among these genres.

Regarding the subjective cognitive and psychological responses, the respondents reported medium levels of perceived social connectedness (mean 2.87 [SD 1.03]) and feelings of security (mean 3.04 [SD 1.11]). And around 80% of respondents reported at least one of the described symptoms of mental disorders. Details of the descriptive results are displayed in Table 2.

**Table 2.** Description of key variables.

Key variables	Mean (SD)	Not at all, n (%)	Rarely, n (%)	Sometimes, n (%)	Often, n (%)	Always, n (%)
Perceived social connectedness (Cronbach α=.92)						
Over the past 14 days of quarantine experience, to what extent have you been bothered by the following:						
(1) I feel disconnected from the world around me.	3.15 (1.24)	53 (8.5)	183 (29.4)	100 (16.1)	190 (30.5)	97 (15.6)
(2) Even around people I know, I don’t feel that I really belong.	2.91 (1.32)	115 (18.5)	139 (22.3)	143 (23)	139 (22.3)	87 (14)
(3) I feel so distant from people.	3.03 (1.33)	96 (15.4)	151 (24.2)	113 (18.1)	164 (26.3)	99 (15.9)
(4) I have no sense of togetherness with my peers.	2.95 (1.27)	106 (17)	132 (21.2)	145 (23.3)	169 (27.1)	71 (11.4)
(5) I catch myself losing all sense of connectedness with society.	2.89 (1.317)	109 (17.5)	162 (26)	125 (20.1)	140 (22.5)	87 (14)
(6) I don’t feel I participate with anyone or any group.	2.85 (1.39)	144 (23.1)	133 (21.3)	103 (16.5)	158 (25.4)	85 (13.6)
Perceived security (Cronbach α=.90)						
Over the past 14 days of quarantine experience, to what extent have you been bothered by the following:						
(1) I am always worried that my life will turn into a mess.	3.23 (1.17)	51 (8.2)	146 (23.4)	99 (15.9)	41.7 (260)	67 (10.8)
(2) I am always worried that my thoughts and emotions will get out of control.	3.08 (1.35)	100 (16.1)	124 (19.9)	144 (23.1)	138 (22.2)	117 (18.8)
(3) I feel that my life is full of uncertainty and unpredictability.	3.26 (1.20)	63 (10.1)	103 (16.5)	158 (25.4)	206 (33.1)	93 (14.9)
(4) I always feel that I am unlucky.	2.96 (1.30)	103 (16.5)	152 (24.4)	113 (18.1)	176 (28.3)	79 (12.7)
(5) I always feel that something will happen to me.	3.10 (1.28)	88 (14.1)	125 (20.1)	137 (22)	180 (28.9)	93 (14.9)
(6) I feel powerless to cope with the unexpected situations in my life.	3.13 (1.28)	81 (13)	133 (21.3)	123 (19.7)	193 (31)	93 (14.9)
Mental well-being (Cronbach α=.88)						
Over the past 14 days of quarantine experience, to what extent have you been bothered by:						
(1) Little interest or pleasure in doing things?	3.14 (1.22)	56 (9)	172 (27.6)	111 (17.8)	196 (31.5)	88 (14.1)
(2) Feeling down, depressed, or hopeless?	2.98 (1.29)	99 (15.9)	137 (22)	152 (24.4)	145 (23.3)	90 (14.4)
(3) Feeling nervous, anxious, or on edge?	3.03 (1.30)	101 (16.2)	127 (20.4)	139 (22.3)	166 (26.6)	90 (14.4)
(4) Not being able to stop or control worrying?	3.04 (1.27)	87 (14)	149 (23.9)	116 (18.6)	193 (31)	78 (12.5)

Hypothesis 1 posited that listening to music from different preferences is associated with the mental well-being of individuals experiencing COVID-19 quarantine. However, our results do not provide evidence to support the hypothesized relationship between music-listening preferences and mental well-being in quarantined individuals, as no significant associations were identified between these variables (pop: *β*=.06, *P*=.34; rock: *β*=−.03, *P*=.88; rap: *β*=−.01, *P*=.19; jazz: *β*=−.02, *P*=.30; quyi: *β*=.01, *P*=.95). Therefore, Hypothesis 1 is not supported.

This study also predicted that engaging with music from different preferences may be linked to the level of mental well-being through the mediation association with perceived social connectedness. Our results showed that the mediation effect varied across different music preferences. Specifically, listening to pop music (*β*=.57, *P*<.001) was positively associated with perceived social connectedness, which, in turn, can be related to a higher level of mental well-being (*β*=.63, *P*<.001). This mediation effect (*β*=.36, *P*<.001) was supported by the bootstrapping approach, with a 95% CI between .2319 and .4971. A similar indirect relationship was also observed in the context of listening to quyi as well (*β*=.20, 95% CI .0499 to .3540; *P*=.009). In contrast, a negative indirect effect, through perceived social connectedness, on the level of mental well-being of individuals was revealed in the preferences for rock (*β*=−.30, 95% CI −.4264 to −.1763; *P*<.001), rap (*β*=−.25, 95% CI −.374 to −.1271; *P*<.001), and jazz music (*β*=−.17, 95% CI −.3133 to −.0359; *P*=.015). Therefore, Hypothesis 2 was partially supported.

**Figure 2.**
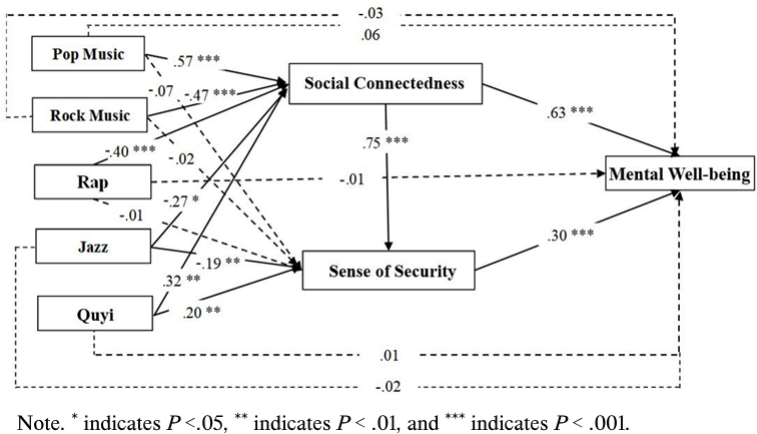
Mediation model.

Hypothesis 3 predicted that listening to music from various preferences may be related to mental well-being through the mediating effect of a sense of security. Our findings indicated that listening to jazz was negatively associated with a sense of security (*β*=−.19, *P*=.003), which, in turn, was related to lower levels of mental well-being (*β*=.30, *P*<.001). The bootstrapping approach supported this mediation effect (*β*=−.06, 95% CI −.1027 to −.0175; *P*=.004). Conversely, a positive indirect effect through a sense of security on mental well-being was also revealed for the consumption of quyi (*β*=.06, 95% CI .0173 to .1060; *P*=.006). However, our results also suggested that there were no significant indirect effects between the consumption of pop (*β*=−0.02, 95% CI −0.0520 to 0.0114; *P*=.276), rock (*β*=.00, 95% CI −.0386 to .0286; *P*=.776), or rap music (*β*=.00, 95% CI −.0372 to .0265; *P*=.804) and mental well-being through a sense of security. Therefore, Hypothesis 3 was partially supported. The details of mediation analysis are presented in Table 3 and Figure 2.

**Table 3.** Mediation results. Age, gender, education, income, and number of vaccines received were controlled for analysis.

Music type	ß	SE	*P* value	95% CI
Model 1 pop music				
Pop music 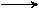 SC^a^ 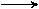 Mental well-being	.36	.07	<.001	.2319 to .4971
Pop music 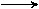 Security 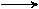 Mental well-being	−.02	.02	.276	−.0520 to .0114
Pop music 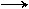 SC 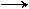 Security 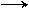 Mental well-being	.13	.03	<.001	.0748 to .1995
Model 2 rock music				
Rock music 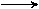 SC 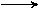 Mental well-being	−.30	.06	<.001	−.4264 to −.1763
Rock music 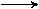 Security 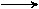 Mental well-being	.00	.02	.776	−.0386 to .0286
Rock music 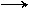 SC 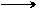 Security 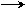 Mental well-being	−.11	.03	<.001	−.1708 to −.0536
Model 3 rap music				
Rap music 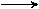 SC 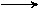 Mental well-being	−.25	.06	<.001	−.3741 to −.1271
Rap music 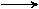 Security 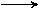 Mental well-being	.00	.02	.804	−.0372 to .0265
Rap music 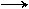 SC 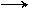 Security 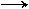 Mental well-being	−.09	.03	<.001	−.1440 to −.0436
Model 4 jazz music				
Jazz music 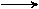 SC 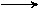 Mental well-being	−.17	.07	.015	−.3133 to −.0359
Jazz music 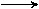 Security 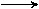 Mental well-being	−.06	.02	.004	−.1027 to −.0175
Jazz music 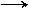 SC 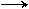 Security 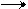 Mental well-being	−.06	.03	.018	−.1197 to −.0114
Model 5 quyi				
Quyi 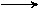 SC 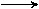 Mental well-being	.20	.07	.009	.0499 to .3540
Quyi 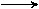 Security 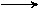 Mental well-being	.06	.02	.006	.0173 to .1060
Quyi 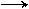 SC 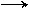 Security 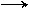 Mental well-being	.07	.03	.012	.0171 to .1409

a SC: social connectedness.

## Discussion

### Principal Findings

Using survey data collected from participants with quarantine experience in mainland China, our study intends to advance knowledge about how different music preferences are associated with individuals’ mental well-being during the COVID-19 quarantine period.

Hypothesis 1 sought to verify whether music preferences were associated with mental well-being in quarantined individuals. However, our findings indicated that preferences for genres such as pop, rock, rap, jazz, and quyi were not found to relate to mental well-being during quarantine. These findings align with a study by Miranda and Claes [43], which also reported no direct relationship between adolescents’ preferences for metal, electronic, and pop music and their levels of depression. The unexpected absence of a predictive relationship may be explained by the study’s control for the number of vaccines participants received, given the high vaccine coverage rate in mainland China. This can be possible, as prior studies also suggest that the association between metal music preferences and higher depression levels in adolescents disappears when other risk factors are accounted for [43,44]. Nonetheless, the causal inference between the 2 key factors remains to be further verified by future experimental and longitudinal studies.

Hypotheses 2 and 3 pertained to the mediation analyses, which posited that music preferences were associated with mental well-being through different intermediate factors. Specifically, our findings revealed a negative indirect relationship between music listening and mental health for certain genres, including rock, rap, and jazz music. That is, preferences for rock, rap, and jazz music were associated with a reduction in individuals’ perceived social connectedness during quarantine, which in turn related to poor mental health. This is partially consistent with previous research, which found that rock and rap music could elicit angry, irritated, and revolted emotional responses and lead to antisocial behavior [45]. A plausible explanation for the negative impact of these music preferences could be the characteristics of these genres, which may facilitate contemplation and reflection during isolation [46]. This contemplation and reflection may enhance feelings of loneliness, thereby making those in quarantine vulnerable to mental problems. In contrast, listening to pop music and quyi during quarantine was associated with higher levels of perceived social connectedness, which, in turn, improved mental well-being. This indicates that both pop music and quyi can fill the emotional void created by the lack of social contact. Quyi, with its cultural and traditional roots, and pop music, which often reflects contemporary social themes, provide listeners with a sense of belonging and emotional attachment, which helps individuals feel part of a larger community, even if physically isolated.

The second pathway of our model highlights a positive association between quyi and individuals’ sense of security, which subsequently contributes to enhanced mental well-being. As security is closely tied to feelings of control and predictability [47], particularly in times of uncertainty, the effect of quyi can be explained by its structured and familiar qualities. These characteristics provide listeners with a feeling of control over their emotions and environment, thereby playing a crucial role in fostering emotional stability. Our finding is also congruent with prior research that reported that people use music listening to gain a sense of security and avoid feeling fear when alone at home [48].

This study has certain practical implications. In the field of health communication and promotion, a large body of research indicates that music consumption is an effective intervention for mental health promotion [35]. By differentiating between various music preferences, our study partially supports this argument for using music consumption to improve mental health, with the evidence indicating that pop music and quyi are indirectly associated with improved mental well-being. This evidence provides insights into the associations between music preferences and levels of mental well-being during quarantine for disease control. Indeed, it is necessary for policy makers and medical professionals to monitor variations in the mental states of quarantined people and understand how these changes may impact the physical health of those individuals. Additionally, professionals should consider the potential effects of music in the quarantine population who should also be aware that the digital consumption of pop music and quyi could be a useful health intervention strategy to build social connection and alleviate the feeling of loneliness.

Furthermore, given the significance of the mediating roles of perceived social connectedness and sense of security in the relationship between music preferences and mental well-being, practitioners are advised to focus more on these factors when supporting individuals in quarantine. For instance, practitioners could provide social support where necessary to create a sense of presence and, thus, enhance the feeling of social connectedness and security, which would lead to better mental outcomes. Overall, the findings of our study serve as a reference for policy makers and practitioners to understand the cognitive and psychological process caused by music consumption during quarantine and develop appropriate health promotion strategies involving music interventions.

### Research Contribution

This paper has a significant research contribution toward the advances in the literature on reactions to environmental stimuli. Specifically, it fills the gap in the research of heterogeneous effects of various music preferences on the mental well-being of quarantine populations during the COVID-19 pandemic. Although the benefit of music listening in mental well-being has been well documented, our findings contribute to the existing literature by demonstrating varying relationships between music preferences and mental well-being through the mediating factors of social connectedness and sense of security. This nuance enhances the understanding of how specific music preference may differentially associate with mental health outcomes in the context of physical isolation.

### Limitations

Despite the practical implications this study provides, it also has several limitations. First, in this study, the potential effect, which pertains to the frequency and duration of exposure to different music preferences and their varying impacts on mental well-being, was not assessed. This omission may lead to an incomplete understanding of the complex relationships between music preference, perceived social connectedness, sense of security, and mental health outcomes. Future research can incorporate detailed measures of music-listening frequency of different genres to elucidate its role more accurately in shaping mental well-being during quarantine conditions.

Second, the nature of the cross-sectional design prevented the this study from assessing the causal relationships between music preference, social connectedness, sense of security, and mental well-being. Therefore, future research could address this limitation by using experimental designs or longitudinal or panel data to explore the intrinsic causal relationships.

Third, music-listening behaviors in this study were measured by responses in a multiple-choice format. Indeed, given that individuals may not be constrained to only 1 music style, the multiple-choice format was applied, and the survey was designed to be concise and clear. Nonetheless, there are also issues with such an approach. For example, the music consumption of various genres was treated as a dichotomous variable (including the values 0 and 1), with a score of 1 indicating that the respondent consumed that style of music. However, this approach may not accurately reflect the respondents’ listening behaviors because of the simplicity of the coding. Future research should apply scale techniques to further explore the intensity and diversity of listening to specific music genres and consider the interplay among different music genres and the impact of this interplay on mental outcomes.

Fourth, another limitation of this study is that perceived social connectedness and security were measured in a general context, rather than specifically in response to music stimuli. This approach was adopted due to the unique challenges posed by the COVID-19 quarantine. Participants may have found it difficult to accurately recall specific emotions or memories related to music listening during that stressful period. The prolonged nature of quarantine, combined with its widespread psychological impact, would have made it challenging to isolate participants’ perceptions directly tied to their music experiences. Therefore, the findings of this study should be interpreted as reflecting associations between music preferences and perceived social connectedness as well as sense of security, rather than establishing any direct or causal relationships. Future research could address this limitation by employing methods that more precisely assess participants’ immediate reactions to music listening, which would allow for a more detailed exploration of the direct effects of music preferences on other variables.

### Conclusions

This study provides empirical evidence that mental well-being is indirectly affected by individuals’ music preferences during quarantine. Specifically, the consumption of pop music and quyi elicits feelings of social connectedness, which, subsequently, enhance feelings of security and, thus, improve psychological well-being. This study offers new insights into previous findings showing that music listening is a prevalent activity during health crisis–induced quarantine, highlighting that the mental health effects of music listening are not uniform and may vary depending on the listening preferences.
